# Advances in rheumatology: new targeted therapeutics

**DOI:** 10.1186/1478-6354-13-S1-S5

**Published:** 2011-05-25

**Authors:** Paul P Tak, Joachim R Kalden

**Affiliations:** 1 Department of Medicine, Division of Clinical Immunology and Rheumatology F4-105, Academic Medical Center/University of Amsterdam, P.O. Box 22700, 1100 DE Amsterdam, The Netherlands; 2 Department of Internal Medicine III (Rheumatology and Immunology), Department for Molecular Immunology, Glueckstrasse 6, 91054 Erlangen, Germany

## Abstract

Treatment of inflammatory arthritides - including rheumatoid arthritis, ankylosing spondylitis, and psoriatic arthritis - has seen much progress in recent years, partially due to increased understanding of the pathogenesis of these diseases at the cellular and molecular levels. These conditions share some common mechanisms. Biologic therapies have provided a clear advance in the treatment of rheumatological conditions. Currently available TNF-targeting biologic agents that are licensed for at east one of the above-named diseases are etanercept, infliximab, adalimumab, golimumab, and certolizumab. Biologic agents with a different mechanism of action have also been approved in rheumatoid arthritis (rituximab, abatacept, and tocilizumab). Although these biologic agents are highly effective, there is a need for improved management strategies. There is also a need for education of family physicians and other healthcare professionals in the identification of early symptoms of inflammatory arthritides and the importance of early referral to rheumatologists for diagnosis and treatment. Also, researchers are developing molecules - for example, the Janus kinase inhibitor CP-690550 (tofacitinib) and the spleen tyrosine kinase inhibitor R788 (fostamatinib) - to target other aspects of the inflammatory cascade. Initial trial results with new agents are promising, and, in time, head-to-head trials will establish the best treatment options for patients. The key challenge is identifying how best to integrate these new, advanced therapies into daily practice.

## Introduction

Recent advances in the treatment of inflammatory arthritides – which include rheumatoid arthritis (RA), ankylosing spondylitis (AS), and psoriatic arthritis (PsA) – have resulted from greater understanding of the pathogenesis of these diseases. Cellular-level and molecular-level research has revealed that these diseases share some common mechanisms [1]. Most critically, the proinflammatory mechanisms of these diseases are associated with progressive joint destruction early in the disease course [2].

In the present article, we review insights into the management of inflammatory arthritides that have been gained from experience with the first generation of TNF inhibitors. We then discuss newer biologic agents as well as novel targeted small molecules that act on signalling pathways, all of which are expanding our knowledge of inflammatory arthritides and providing more comprehensive management options.

## Lessons learned from TNF inhibitors

The development of biologic agents that selectively block cytokines has provided a major advance in the treatment of inflammatory arthritides [3,4]. TNF is a proinflammatory cytokine known to be present in higher concentrations in patients with RA, AS, and PsA. This cytokine plays a dominant role in the inflammatory cascade under lying various inflammatory disorders [5-8]. TNF is both an autocrine stimulator and a potent paracrine inducer of other inflammatory cytokines, including the interleukin family [8].

To date, three TNF-targeting agents have dominated the biologic management of RA, AS, and PsA. Etanercept, a dimeric fusion protein, consists of the extracellular portion of the human p75 TNF receptor linked to the Fc region of human IgG_1_[9,10]. Infliximab, a chimeric human–murine monoclonal antibody, binds to TNF and consists of human constant and murine variable regions. Adalimumab is a recombinant human monoclonal antibody specific to TNF [11,12]. All three anti-TNF therapies have well-demonstrated efficacy in RA, AS, and PsA [9,11,12]. This section focuses on these three agents, for which the most data exist.

In RA (for which most data have been accrued), early treatment with any one of these antagonists in combination with methotrexate (MTX) leads to low disease activity or remission in a considerable percentage of patients [13-15]. TNF inhibitors can potentially prevent radiological progression and thereby prevent disability. However, the pharmacokinetics and binding profiles of these agents are different [1]. Nevertheless, randomised clinical trials (RCTs) in RA strongly suggest that all three TNF inhibitors effectively reduce signs and symptoms, improve physical function, and inhibit progression of structural damage.

According to the manufacturers, an estimated 1,136,000 patients have been exposed to Infliximab, 500,000 patients to etanercept, and 370,000 patients to adalimumab worldwide since these products became commercially available. The regular monitoring requirements for TNF inhibitors are less stringent than those required for many conventional disease-modifying antirheumatic drugs (DMARDs). TNF inhibitors are commonly used in combination with conventional DMARDs, however, so most patients will still require monitoring.

### Safety

Bacterial infections, including sepsis and pneumonia, invasive fungal infections, and other opportunistic infections (for example, pneumocystosis, candidiasis, listeriosis, aspergillosis), have been reported with the use of TNF inhibitors [9,11,12]. Reactivation of latent tuberculosis following treatment has led to the introduction of pre-initiation screening procedures, which have successfully reduced the number of reported cases [16,17]. The risk of reactivation of latent tuberculosis is, of course, dependent on the incidence of latent infection and is associated with all TNF inhibitors [18,19]. Some registry data, however, suggest that the risk may be lower with etanercept [20-22]. In RA patients, risk factors include active longstanding disease, age, country of origin, history of exposure to a person with tuberculosis, concomitant use of immunomodulators, and disease activity [23]. Physicians should remain alert to the development of symptoms related to tuberculosis or other infections.

Owing to adverse effects observed during clinical trials, patients with congestive heart failure should be closely monitored if they are receiving TNF inhibitors [9,11,12]. Other rarely reported conditions possibly related to use of TNF inhibitors include demyelinating disease, seizures, aplastic anaemia, pancytopaenia, and drug-induced lupus [9,11,12]. Physicians should remain vigilant for the development of these conditions [16].

### Formation of antibodies

The formation of antibodies to biologic agents is a significant issue because antibodies have the potential to reduce the efficacy of the agent or to cause adverse events [10]. All three TNF inhibitors have been associated with the development of antibodies, although etanercept does not appear to generate neutralising antibodies [9,10,10,12,24-26]. The use of MTX in combination with TNF inhibitors appears to reduce the incidence of antibody formation [10-12,24].

In a cohort study of 53 patients receiving etanercept for AS without MTX, mean etanercept levels in responders and nonresponders at 12 and 24 weeks were similar, and no antibodies to etanercept were detected [27]. No correlation was found among etanercept levels, formation of antibodies to etanercept, and clinical response. Conversely, in a 54-week cohort study of 38 patients receiving Infliximab for AS, detection of antibodies to Infliximab was associated with undetectable serum trough Infliximab levels and reduced response to treatment [28].

### Shared mechanisms

A look at the cellular and molecular levels of diseases in rheumatology demonstrates that such diseases share common mechanisms and may be more closely related than previously recognised. Rigorous studies have examined the mechanisms of action of the anti-TNF inhibitors, particularly Infliximab and etanercept; however, many questions remain unresolved [1]. For example, although both Infliximab and etanercept are useful in the treatment of peripheral arthritis and AS, there appear to be differences in their effects at the cellular level [29,30]. Moreover, while their actions in AS have yet to be fully elucidated, the long-lasting suppression of T-cell function apparent during treatment with Infliximab suggests that neutralisation of soluble TNF cannot be the only mechanism [29]. Possible mechanisms generally fall into two categories: those mediated by blockade of the TNF receptor, and those mediated by induction of trans-membrane TNF. Several mechanisms probably act simultaneously.

To what extent various mechanisms contribute to drug efficacy remains an open question. All of the anti-TNF agents bind to transmembrane TNF and could theoretically induce both complement-dependent cytotoxicity and antibody-dependent cellular cytotoxicity, although at lower levels for etanercept compared with the anti-TNF agents Infliximab and adalimumab [1]. The roles of apoptosis and inflammation reversal for reducing cellularity in rheumatoid synovial tissue during anti-TNF therapy are unclear [1]. A study by Wijbrandts and colleagues analysed apoptosis in peripheral blood and synovial tissue within 24 hours of treatment with Infliximab in patients with RA. There were no signs of apoptosis induction in peripheral blood monocytes or lymphocytes after Infliximab treatment. These results support the view that the rapid decrease in synovial cellularity observed after initiation of anti-TNF therapy cannot be explained by apoptosis induction at the site of inflammation [31].

### Routes of administration

The TNF inhibitors all require parenteral administration, either intravenously (Infliximab) or via subcutaneous injection (etanercept, adalimumab) [4]. The availability of different formulations allows tailoring of treatment to the individual and ensures that the patient is receiving maximal benefit with minimal negative impact on their quality of life. Although some patients appreciate the control offered by self-administration of subcutaneous injections, others do not like to self-inject. Intravenous drugs can be inconvenient because of the need for regular hospital visits, but some patients desire regular contact with medical professionals. The decision on whether to use an intravenous or subcutaneous product should be based on the clinician’s and patient’s goals for treatment.

Intravenous administration allows high serum concentrations to be rapidly achieved, and therefore offers the potential for fast, complete suppression of inflammation [32,33]. Rapid improvement in signs and symptoms has been observed following the usual clinical dose of Infliximab (3 mg/kg) in RA patients [34]. Within 48 hours of administration, patients experienced significant improvements in the mean duration of morning stiffness, patient assessment of pain, physician global assessment of arthritis, and patient global assessment of arthritis compared with baseline measurements. Studies using a high-dose infusion of Infliximab (10 mg/kg) in RA patients have shown significant reductions in C-reactive protein levels [35,36], improvements in Disease Activity Score (DAS) and American College of Rheumatology (ACR) response [37], and significant reductions in bone resorption as measured by β-CrossLaps, a predictor of annual bone loss in RA, as soon as 24 hours post infusion [37]. The benefits of higher doses, however, must be weighed against accompanying increases in side effects. Additionally, Infliximab therapy has demonstrated a reduction in the number of inflammatory cells, including intimal and sublining macrophages, T cells, and plasma cells, in rheumatoid synovial tissue as soon as 48 hours after initiation of treatment [33]. Although unlicensed, intravenous administration of adalimumab also has demon strated a rapid onset of clinical effect [38]. Whether intravenous administration of TNF antagonists has a faster effect than subcutaneous administration is not known presently, as no direct comparisons have been published.

Subcutaneous agents may be appropriate for and preferred by some patients. Although drug absorption into the bloodstream is slower and a delay of several days is possible before maximal concentrations are reached, desired outcomes can be achieved. While a rapid onset of effect for intravenous administration has been established, there is on average no clear-cut difference in long-term overall efficacy outcomes between subcutaneous and intravenous administration.

### Unmet needs in biologic therapy with TNF inhibitors

Although TNF inhibitors are currently the gold standard of biologics for patients with inflammatory arthritides, there are still a number of outstanding questions regarding how to gain the maximum benefit from these agents. The most recent ACR guidance stating that patients with early RA are not candidates for biologic therapy [18] is debatable. There are convincing data indicating that the use of biologics early in the course of the disease can be highly efficacious and may induce clinical remission in a certain percentage of patients [13,15,39-41]. Additional data may spur modification of guidelines and practice for those early RA patients who do not respond sufficiently to conventional treatment. Of importance, a well-defined referral pathway within health care systems is needed to identify patients early in the course of the disease. Also, family physicians and other healthcare professionals must be educated about the early symptoms of inflammatory arthritides, with an emphasis on the importance of early referral to rheumatologists for diagnosis and treatment [42].

Likewise, additional studies are needed to determine whether patients with co-morbidities or those taking concurrent medications require monitoring for specific toxicities [4]. Several registries have reported a high prevalence of co-morbid conditions in RA patients who are commencing biologic therapy in routine practice [43,44]. Oldroyd and colleagues compared 354 patients with AS from the Australian Rheumatology Association Database who were commencing biologic therapy with more than 1,000 enrolees from four RCTs involving biologic therapy. At baseline, patients from the Australian Rheumatology Association Database – considered representative of the general population seeking clinical care – were found to have much higher levels of co-morbidity than the RCT subjects, as well as significantly greater disease activity. These findings have important implications for patient monitoring [45].

In a broader sense, RA trial inclusion criteria may need to be less restrictive [46]. A comparison of 546 RA patients from the Dutch Rheumatoid Arthritis Monitoring registry with 1,223 RA patients from 11 RCTs showed much greater disease activity at baseline in RCT enrolees [47]. The efficacy of TNF-blocking agents was lower in Dutch Rheumatoid Arthritis Monitoring registrants. For example, in 10 of the 11 comparisons, the ACR 20% improvement criteria (ACR20) response rate was lower in the registry cohort (again, representative of daily clinical practice) than in the RCT group, and the difference was significant in five of the 11 comparisons. These data indicate a smaller, real-world effect of anti-TNF treatment than the effect seen in trials. The discrepancy may be due to continued use of co-medication and selection toward greater disease activity in RCTs.

Zink and colleagues obtained similar results during their comparison of 1,458 patients from the Rheumatoid Arthritis Observation of Biologic Therapy registry with data from five major RCTs that led to approval of biologics for RA. Only 21 to 33% of Rheumatoid Arthritis Observation of Biologic Therapy registrants would have been eligible for the trials, and this ineligible group demonstrated lower TNF inhibitor response rates than RCT enrolees who received biologic therapy. The investigators concluded that observational cohort studies, which include a full spectrum of patients (for example, with various co-morbidities, taking assorted concomitant medications), are essential to complement RCT data [46]. A study of 417 RA patients from the Danish Database for Biological Therapies in Rheumatology further supports these clinical practice data. In the majority of these routine care patients, TNF antagonists were not successful in controlling disease, although they did achieve moderate overall success in controlling clinical inflammation [48]. Clearly, a bridge is needed between trial results and real-world results.

Some studies have hypothesised that TNF inhibitors may have the potential to repair RA joint damage [49,50]. The data to support this notion are currently negligible, however, and tools to measure and evaluate repair must be developed before in-depth investigations can be launched.

### Potential for effectiveness of TNF antagonists in early rheumatoid arthritis

In one study, a small number of patients experiencing RA symptoms for <12 months but considered to have a poor prognosis were randomised to receive either Infliximab plus MTX (*n* = 10) or placebo plus MTX (*n* = 10) for 1 year [51]. Patients receiving Infliximab experienced significant improvements in all measures at the end of year 1 compared with those receiving placebo. The Infliximab patients then received MTX alone for an additional year, and 70% of patients maintained the Infliximab responses, as measured by the C-reactive protein level, DAS in 28 joints (DAS28), and Health Assessment Questionnaire results [51].

van der Kooij and colleagues recently compared the clinical and radiological efficacy of initial (*n* = 117) versus delayed (*n* = 67) treatment with Infliximab plus MTX in patients with early RA in a *post hoc* analysis of the BeSt study [52]. After 3 years of treatment, patients receiving initial Infliximab plus MTX demonstrated more improvement in functional ability over time, as measured by the Health Assessment Questionnaire, and were less likely to have radiological progression than patients treated with delayed Infliximab plus MTX. These results suggest that initial treatment with a biologic-plus-DMARD combination in patients with recent-onset RA is more beneficial than reserving such treatment for patients in whom traditional DMARDs have failed [52].

The PREMIER study compared the efficacy of early intervention with a combination of adalimumab and MTX versus either agent used alone as monotherapy in patients with early, aggressive RA [15]. The primary end points in this 2-year, double-blind, controlled study (*n* = 799) were the percentage of patients in whom an ACR50 response was achieved and the mean change from baseline in the modified Total Sharp Score, which assesses bone erosion and joint space narrowing on radiographs. Combination therapy was superior to adalimumab and MTX mono therapy in all outcomes measured. At year 1, patients treated with combination therapy had a mean increase in Total Sharp Score of 1.3 units compared with 3.0 units in those receiving adalimumab monotherapy (*P* = 0.002) and of 5.7 units in those receiving MTX monotherapy (*P* <0.001). At year 2, patients receiving combination therapy continued to have significantly less radiographic progression (mean change 1.9 Sharp units) compared with those treated with either adalimumab (5.5 units) or MTX (10.4 units) monotherapy (*P* <0.001 for both comparisons). Although ACR responses were comparable in the two monotherapy arms, there was significantly less progression in the adalimumab arm compared with the MTX arm at 6 months (2.1 vs. 3.5), 1 year (3.0 vs. 5.7) and 2 years (5.5 vs. 10.4) (*P* <0.001 for all comparisons). This is another study suggesting the value of combination therapy in early RA [15].

Van der Heijde and colleagues have hypothesized that therapeutic intervention early in the disease course has a disproportionate benefit on outcome if treatment is started early in the disease course [51]. Additionally, drug-free remission may be a realistic goal in some patients with early RA. In the BeSt study, 19% of patients who received Infliximab plus MTX in a DAS-steered, tightly controlled manner were in drug-free remission at 5 years, for a mean duration of 22 months. Infliximab had been successfully discontinued in 58% of patients, while 18% were still receiving combination therapy. Furthermore, compared with other treatment strategies, initial temporary treatment with Infliximab plus MTX resulted in significantly better functional ability over 5 years [53]. These studies raise the possibility that if aggressive treatment to induce remission is instituted very early in the course of RA, more conservative management strategies may be sufficient to maintain that remission.

The use of TNF blockers for early-stage PsA is currently under discussion. For early-stage AS, one study showed Infliximab to be highly efficacious in patients who were positive for HLA-B27, had recent-onset inflammatory back pain, and had early sacroiliitis demonstrated by magnetic resonance imaging [54].

### Prediction and discontinuation of TNF antagonists

Additional unmet needs include: the ability to predict clinical response so that these drugs, which are expensive and have the potential for serious toxicity, can be targeted to patients who would most benefit [55]; an understanding of acquired drug resistance to anti-TNF agents [56]; a full explanation for why patients with spondyloarthritis (a group of disorders that includes AS and PsA) have a 20% lower probability of discontinuing TNF antagonists than patients with RA [57]; and an understanding of reasons for and predictors of discontinuation.

Relative to the first point, the search for predictors of response is important in the context of personalised medicine, with the aim of increasing the percentage of patients exhibiting a robust response to a given treatment. Wijbrandts and colleagues recently studied arthroscopic synovial tissue in 143 patients with active RA prior to initiating treatment with Infliximab [58]. Their analysis confirmed that the baseline level of TNF expression may be a significant predictor of response to anti-TNF therapy. At baseline, TNF expression in the intimal lining layer and synovial sublining was significantly higher in responders than in nonresponders (clinical response determined at week 16) (*P* = 0.047 and *P* = 0.008, respectively). The number of macrophages, macrophage sub sets, and T cells was also significantly higher in responders than in nonresponders [58]. The relationship between synovial lymphocyte aggregates and the clinical response to Infliximab has also been studied in RA patients [59]. Synovial tissue biopsy samples were obtained from 97 patients with active RA before initiation of Infliximab treatment. Lymphocyte aggregates were counted and graded for size, and logistic regression analysis identified whether the presence of lymphocyte aggregates could predict clinical response at week 16. The majority (57%) of RA synovial tissues contained lymphocyte aggregates. Additionally, aggregates were found in 67% of clinical responders compared with 38% of nonresponders. The presence of aggregates at baseline was a highly significant predictor of the clinical response to anti-TNF treatment (*P* = 0.008), demonstrating that RA patients with synovial lymphocyte aggregates may have a better response to Infliximab treatment than those with only diffuse leucocyte infiltration [59].

Relative to the fourth point, 21 to 35% of patients discontinue TNF-blocking agents within the first year [60]. Reasons for discontinuation appear to include lack of response, loss of response, development of intolerance, partial efficacy, and adverse events [61,62]. Switching to a different TNF inhibitor may be an option for some patients [63]. One limited study with 31 enrolees suggested that when etanercept is not efficacious, Infliximab may offer gains, and that when Infliximab fails due to adverse events, etanercept may allow continuation [61]. Another larger study (complete data for 197 patients) in RA suggested that a second TNF inhibitor may be effective after failure of the first inhibitor, regardless of the reason for discontinuation of the first agent [60]. Conceivably, efficacy of a second TNF blocker may be lower in primary nonresponders to a first TNF blocker (response being defined at 12 to 16 weeks after initiation of treatment). Switching to a different mechanism of action and agent, such as rituximab, abatacept, or tocilizumab, is also an option (see below).

Identifying predictors of discontinuation would be valuable in managing disease and targeting therapies to patients most likely to benefit. Currently, treatment choices are dominated by patient and physician preference, side-effect profiles, and cost [64]. A cohort (*n* = 503) from the Brigham Rheumatoid Arthritis Sequential Study was examined to identify clinical predictors associated with discontinuation of TNF inhibitors [64]. In this study, 210 out of 503 patients (42%) discontinued therapy. Unfortunately, only 63 patients gave a reason; the investigators therefore shifted to a model-based analysis. The results showed that higher risk of discontinuation was associated with prior use of another TNF agent. Lower risk of discontinuation was associated with longer disease duration, prior use of DMARDs, and longer MTX use.

More information is clearly needed with regard to individualising physician/patient decision-making about initiating anti-TNF agents, switching agents, and predicting efficacy and tolerability. Lowering the discontinuation rates is an important current goal.

## Newly discovered mechanisms of action

**Figure 1:**
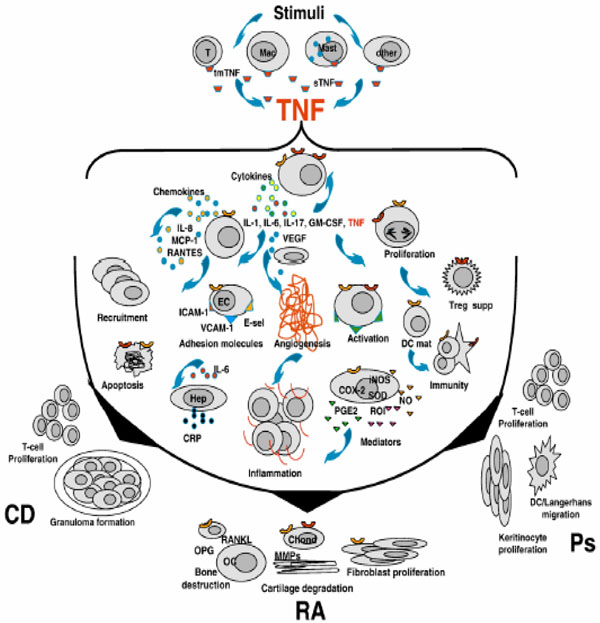
**Relationships between cytokines.** The cascade and network of cellular responses mediated by TNF common to inflammatory arthritides: rheumatoid arthritis (RA), Crohn’s disease (CD) and psoriasis (Ps). Chond, chondrocyte; COX-2, cyclooxygenase-2; CRP, C-reactive protein; DC, dendritic cell; EC, endothelial cell; E-sel, E-selectin; GM-CSF, granulocyte–macrophage colony-stimulating factor; Hep, hepatoxyte; ICAM-1, intercellular adhesion molecule-1; IL, interleukin; INOS, inducible nitric oxide synthase; Mac, macrophage; MCP-1, monocyte chemotactic protein-1; MMP, matrix metalloproteinase; NO, nitric oxide; OC, osteoclast; OPG, osteoprotegerin; PGE2, prostaglandin E_2_; RANKL, receptor activator of NF-κB ligand; RANTES, regulated on activation normal T-cell expressed and secreted; ROI, reactive oxygen intermediates; SOD, superoxide dismutase; Treg supp, suppression of T regulatory cells; VCAM-1, vascular cell adhesion molecule-1; VEGF, vascular endothelial growth factor. Reproduced with permission from [1].

More than 100 cytokines and chemokines have been identified in the inflammatory cascade associated with inflammatory arthritides [1]. Although TNF is a key player in the proinflammatory cytokine cascade, the complex interconnectivity and dynamics of cytokine biology mean that relationships between cytokines may be better visualised as a network within a cascade (Figure 1) [1,65].

Increased understanding of the pathophysiology of RA has led to the identification of new therapeutic targets, including proinflammatory cytokines, T cells and B cells, adhesion molecules, chemokines, and intracellular and extracellular signalling pathways. The first stage in the pathogenesis of RA is thought to be the activation of T cells via the T-cell receptor complex [66]. The second stage involves interaction between co-stimulatory molecules on T cells and molecules on antigen-presenting cells, providing more targets for intervention [66]. Fibroblast-like synoviocytes are resident mesenchymal cells of the synovial joints and are increasingly recognised as key players in the pathogenesis of RA. Activation of fibroblast-like synoviocytes produces a broad array of cell surface and soluble mediators that help to recruit, retain, and activate cells of the immune system and resident joint cells, leading to the promotion of ongoing inflammation and tissue destruction [67].

Cytokines such as IL-6, IL-12, IL-15, IL-17, IL-18, IL-21, IL-23, IL-33, and IFNγ provide potential targets for modulation [68], as do the signal transduction systems that follow the binding of cytokines to cell receptors, typically sequences of protein kinases such as mitogen-activated protein kinase [69]. Factors that modulate the transcription of genes following cytokine stimulation, such as NF-kB, provide more targets for modulation of cytokine pathways [70,71].

B cells are also important in the pathophysiology of RA, although their role is not as well understood as that of T cells. B cells produce autoantibodies, may act as antigen-presenting cells, secrete proinflammatory cyto-kines such as IL-6, and regulate T cells. In addition to possibly acting as antigen-presenting cells, B cells produce immunoglobulins and secrete cytokines, perpetuating inflammation. Depletion of B cells is a logical therapeutic strategy that should provide a reduction in immuno-inflammatory components [72,73]. B-cell-related potential targets include B-lymphocyte stimulator and the proliferation-inducing ligand APRIL. Both assist the survival, proliferation, and antigen presentation of B cells. An exploratory phase IB trial of the recombinant fusion protein atacicept, which binds and neutralises B-lymphocyte stimulator and APRIL, was recently completed [74]. B cells also exhibit a regulatory capacity by controlling dendritic cell and T-cell function through cytokine production [75,76]. B-cell signalling pathways are emerging as potential therapeutic avenues. Targets include Bruton tyrosine kinase, which plays a key role in B-cell development and activation, and B-lymphocyte stimulator, which is important to B-cell survival and maturation [77].

Autoantibodies, such as anticitrullinated peptide antibodies and rheumatoid factor, serve as diagnostic and prognostic markers of RA. Their presence in a variety of autoimmune diseases suggests that they may also be valuable therapeutic targets. For example, blockade of B-cell trafficking may inhibit formation of autoantibodies [77]. This is an area ripe for investigation.

Other areas of research include modulating complement activation to prevent the influx of inflammatory cells into the synovium and inhibiting chemokines [78] to prevent the degradation of cartilage and bone [66]. The receptor activator of NF-κB/receptor activator of NF-κB ligand pathway is also being targeted with the aim of regulating the formation and activation of osteoclasts [79].

Lastly, although it is still unclear whether patients who fail one TNF blocker should switch to another TNF blocker or to a drug with a different mechanism of action, in RA in the recent past it has been common to try another TNF blocker after treatment with the first TNF blocker has failed [80]. However, it is possible that TNF is not the crucial cytokine instigating RA in primary nonresponders (patients with no response 12 to 16 weeks after initiation of therapy) to anti-TNF therapy [58,80]. Initial evidence that primary nonresponders are less likely to respond to a second TNF blocker may accelerate the search for non-TNF targets [80]. Consistent with this notion, lower synovial TNF expression and fewer TNF-producing inflammatory cells are, on average, present in primary nonresponders [58]. Pharmacokinetics and pharmacogenetics are expected to elucidate these concepts [81].

## Advances in biologic therapy

There are many agents in development for the treatment of inflammatory arthritides. This is a highly competitive arena due to the complexity of interrelated pathways contributing to inflammatory arthritis pathogenesis [66]. Establishing the exact role of different treatments and identifying which patients will benefit most from them are the challenges now facing rheumatologists.

### Rituximab

Rituximab, a chimeric anti-CD20 monoclonal antibody, was the first B-cell agent approved for treatment of RA [82]. This antibody was approved in combination with MTX in the United States and Europe in 2006 for adult patients with, respectively, moderate to severe active RA or severe active RA, after the failure of at least one TNF inhibitor. The agent targets B cells, rather than the entire immune system, and is administered by intravenous infusion to patients with an inadequate response to TNF inhibitors [83]. Rituximab has been shown to inhibit progression of structural damage in RA over 2 years, and continues to inhibit joint damage with long-term treatment [39,84].

In the event of inadequate efficacy with a TNF inhibitor, some have suggested that switching patients to rituximab is a more effective management strategy than switching to another TNF inhibitor [85]. A prospective cohort study of 318 RA patients found that when the motive for switching to rituximab was TNF inhibitor ineffectiveness, disease improvement was significantly better than with an alternative TNF inhibitor [85]. If the reason for switching is not lack of efficacy (for example, adverse events, patient preference), there is no advantage in switching to rituximab [85].

Immunoglobulin levels have been found to be lower in patients receiving rituximab in the long term for RA [86]. An initial apparent trend toward higher rates of serious infection in this population may have been discounted by an open-label study of 1,039 RA patients [87]. The serious infection rate was 5.0 per 100 patient-years, similar to that for etanercept, Infliximab, and adalimumab (5.3 per 100 patient-years) [88]. There also have been reports of psoriasis and PsA developing in RA patients receiving rituximab [89]; however, the same is true for TNF inhibitors [90]. The development of progressive multifocal leukoencephalopathy or hepatitis B reactivation during rituximab treatment for RA is very rare.

### Abatacept

Abatacept is a T-cell co-stimulation modulator administered by intravenous infusion. The modulator is thought to prevent the activation of T lymphocytes, including naïve T cells [91,92]. Abatacept was approved in the United States and Europe in 2005 for treatment of RA in adult patients with an inadequate response to DMARDs or TNF inhibitors. In January 2010 it was approved in Europe for moderate-to-severe active polyarticular juvenile idiopathic arthritis in patients 6 years of age and older. Because abatacept was the first therapy targeting the inhibition of co-stimulatory signals to prevent T-cell activation, its use in early disease [93] and in biologic-naïve patients with active RA [94] has generated particular interest and investigation [91,95-97]. These data may support the use of abatacept in biologic-naïve patients with early disease who have had an inadequate response to MTX.

The magnitude of abatacept’s effect appears to increase over time. According to the initial report of the Abatacept in Inadequate Responders to Methotrexate, Abatacept or Infliximab versus Placebo, a Trial for Tolerability, Efficacy, and Safety in Treating Rheumatoid Arthritis study, clinical response and disease activity were not only maintained from 6 to 12 months, but also appeared to improve [98]. The report containing 2-year results is currently only in abstract form but shows that reduced disease activity was maintained with ongoing abatacept treatment [94,99]. Abatacept has also demonstrated an increasing and significant degree of inhibition of structural damage progression in patients receiving treatment for 2 years [95]. Abatacept may have an increasing disease-modifying effect on structural damage over time in the majority of patients who respond to treatment. To date, this is a unique observation among biologic treatments for RA.

The long-term efficacy and safety of abatacept have been demonstrated over 5 years with a dose of 10 mg/kg [97]. In a long-term extension trial, abatacept was well tolerated and provided durable improvements in disease activity, with no unique safety events reported. These data, combined with relatively high retention rates, confirm that abatacept provides sustained clinical benefits in RA. Additionally, abatacept has been shown to provide clinical benefits in patients with RA who have previously failed TNF inhibitor treatment, regardless of the previous TNF inhibitor(s) used or the reason(s) for treatment failure [100]. This finding suggests that switching to abatacept may be a useful option for patients who fail TNF inhibitor treatment.

### Tocilizumab

Tocilizumab is a humanised anti-IL-6-receptor monoclonal antibody administered by intravenous infusion. This antibody inhibits signals through both membrane and soluble IL-6 receptors [101]. Tocilizumab has received approval in Europe and the United States (January 2009 and 2010, respectively) for the treatment of moderate to severe RA in adult patients who have responded inadequately or have been intolerant to previous therapy with one or more DMARDs or TNF antagonists.

Tocilizumab used as monotherapy or in combination with MTX has demonstrated superiority over MTX mono therapy in reducing disease activity in RA over 24 weeks [102,103]. Furthermore, tocilizumab has resulted in significant improvements compared with placebo in physical function, fatigue, and physical and mental health scores over 24 weeks in patients who fail to respond to conventional DMARD therapy alone [104]. Tocilizumab has also demonstrated efficacy in RA patients who fail to achieve an adequate response with or became refractory to TNF inhibitors [105].

There is a close relationship between normalisation of serum IL-6 levels following treatment with tocilizumab and clinical remission. In the phase III SATORI trial, patients whose serum IL-6 levels became normal tended to achieve DAS28 remission. Normal IL-6 levels may therefore provide a good marker to identify patients who can stop tocilizumab treatment without the risk of flaring [106,107].

In the 3-year extension of the SAMURAI study, patients with early RA treated with tocilizumab exhibited strongly suppressed radiographic progression [108]. Further more, radiographic progression was more effectively suppressed in patients who received tocilizumab at the start of the trial than in those who received conventional DMARDs at the start. Early introduction of tocilizumab treatment may therefore be more effective in preventing joint damage. The LITHE study in 1,196 patients who had inadequate responses to MTX further supports the potential for tocilizumab to suppress radiographic progression [109]. Patients also demonstrated improvements in physical function.

Tocilizumab has a well-characterised safety profile, with infections being the most common adverse event in trials [101,109]. Safety data pooled from five pivotal tocilizumab studies demonstrate rates of serious infection of 3.5 per 100 patient-years for the 4 mg/kg dose and of 4.9 per 100 patient-years for the 8 mg/kg dose compared with 3.4 per 100 patient-years for the comparator groups over a median 3.1 years’ treatment duration [109]. Physicians should also monitor for decreased neutrophil counts and increased lipid or liver enzyme levels, and manage appropriately [101,109].

### Certolizumab pegol

Certolizumab is a pegylated Fab fragment of a humanised anti-TNF monoclonal antibody that neutralises the activity of TNF [66]. Certolizumab was approved for treatment of RA in combination with MTX in the United States and Europe in 2009. The use of pegylation increases the half-life of the molecule and eliminates the chimeric Fc portion. It is therefore hoped that adding poly ethylene glycol will produce a longer-lasting compound with fewer side effects, although it remains to be established whether pegylation does indeed confer these advantages in clinical practice [66].

Subcutaneous administration of 400 mg certolizumab every 4 weeks as monotherapy has demonstrated a rapid onset of response and reduction in RA disease activity as early as week 1 [110]. When used in combination with MTX, certolizumab (400 mg at baseline weeks 2 and 4, then 200 or 400 mg every 2 weeks) reduces radiographic progression compared with MTX alone over 1 year, and the difference is already significant at 6 months [111].

### Golimumab

Golimumab is a fully human anti-TNF IgG_1_ monoclonal antibody that targets and neutralises both the soluble and membrane-bound forms of TNF [66]. Golimumab was recently approved for monthly subcutaneous treatment of adults with RA, PsA, and AS. A randomised, double-blind, placebo-controlled dose-ranging study compared subcutaneous injections of golimumab with placebo in patients with active RA despite treatment with MTX [112]. In this study, greater efficacy was demonstrated for golimumab 50 mg every 4 weeks in addition to MTX compared with MTX plus placebo in terms of ACR responses. Furthermore, 20% of patients receiving golimumab achieved DAS28 remission at week 16, compared with only 5.7% (*P* = 0.074) of patients receiving MTX alone. Over a 52-week treatment period, all clinical responses achieved at week 16 were maintained and/or improved, and no unexpected safety issues were observed [112].

These results have been further confirmed in a phase III study in patients with established RA and disease activity despite treatment with MTX monotherapy [113]. Additionally, golimumab demonstrated efficacy in patients with established RA who had previously received other TNF inhibitors and in MTX-naïve patients [114,115].

Efficacy has also been demonstrated in patients with PsA and AS treated with golimumab [116], similar to that for currently available TNF inhibitors [117,118]. Furthermore, golimumab is capable of increasing function in patients with AS [118]. In PsA, golimumab has also demonstrated improvements in psoriatic skin and nail disease [116].

### Ustekinumab

Ustekinumab is a human monoclonal antibody directed against the p40 subunit of IL-12/IL-23 that has demonstrated efficacy in PsA [119]. In a parallel-group crossover study involving 146 patients, a significantly higher proportion of ustekinumab-treated patients achieved a response using ACR criteria compared with placebo-treated patients at week 12. Ustekinumab was approved in 2009 in both the United States and Europe for treatment of patients with moderate-to-severe plaque psoriasis. Ustekinumab has not been approved for PsA.

## Kinase targets in development

Kinases such as Janus kinase 3 are intracellular molecules that play a pivotal role in signal transduction of interleukins. CP-690550 is an oral Janus kinase inhibitor developed to interfere with these enzymes. In a recent study, 264 patients were randomised equally to receive placebo, 5 mg CP-690550, 15 mg CP-690550, or 30 mg CP-690550 twice daily for 6 weeks and were followed for an additional 6 weeks after treatment. The primary efficacy endpoint was the ACR20 response rate at 6 weeks [120]. Response rates were 70.5%, 81.2%, and 76.8%, respectively, in the groups receiving 5 mg, 15 mg, and 30 mg CP-690550 twice daily compared with 29.2% in the placebo group (*P* <0.001). This study also assessed pain, physical functioning, and health status using 100-mm visual analogue scales, the Health Assessment Questionnaire – Disability Index, and the self-administered Short-Form 36 [121]. Treatment with CP-690550 resulted in clinically meaningful and statistically significant patient-reported improvements by week 1 of treatment. The incidence of blood lipid elevations and neutropaenia is concerning, however, and much longer-term studies are needed.

Also of interest are data indicating that spleen tyrosine kinase could serve as a novel and promising target for immune intervention in rheumatic diseases. R788, a novel and potent small-molecule spleen tyrosine kinase inhibitor, recently demonstrated the ability to ameliorate established diseases in lupus-prone NZB/NZW F1 mice and MRL/lpr mice, and also significantly reduced clinical arthritis in collagen-2-induced arthritis models [122,123]. In a recent 12-week double-blind study, 142 patients with active RA despite MTX therapy received R788 at concurrent doses of 50 mg, 100 mg, or 150 mg twice daily; 47 patients received MTX plus placebo [124]. The primary endpoint, an ACR20 response at week 12, was achieved by the majority of patients receiving 150 mg or 100 mg twice daily (72% vs. 65%; *P* <0.01). Around one-half of the patients achieved an ACR50 response (57% vs. 49%), and more than one-quarter of patients achieved an ACR70 response (40% vs. 33%). These results suggest that spleen tyrosine kinase inhibition is worthy of more in-depth study.

## Conclusion

New approaches to inflammatory arthritides are challenging the rheumatologist. The advent of biologic therapies has revolutionised treatment and has allowed us to further influence the progression of these diseases as well as their symptoms. Development of the first biologics, TNF inhibitors, expanded our knowledge of the pathogenesis of inflammatory conditions. As TNF inhibitors have been available to rheumatologists for more than a decade, a large body of data has accumulated regarding their safety and efficacy. More recently, biologics with a distinct mechanism of action (rituximab, abatacept, and tocilizumab) have been approved. Numerous other targets within the inflammatory cascade continue to be identified, and biologic and nonbiologic agents to modulate/inhibit the associated pathways are either in the pipeline or have already been developed. The relative efficacy of these agents remains to be established, and, in time, head-to-head trials will be required to determine the best treatment options for patients.

An international task force comprising more than 60 rheumatology experts and a patient recently developed recommendations for achieving optimal therapeutic outcomes in RA. Using a Delphi-like procedure, the members discussed, amended, and voted on evidence derived from a systematic literature review as well as expert opinion. The resulting initiative, called Treat-to-Target, shares information and strategies in an effort to determine the best options for patients [125].

In the meantime, the prospect of preventing radiographic damage has led to a re-evaluation of how patients with inflammatory arthritides are managed, with early diagnosis and referral becoming increasingly important. Additionally, researchers are acknowledging specific subgroups of patients who are more likely to derive benefit from certain treatments. Before offering treatment options, the rheumatologíst needs to be able to identify patients who are likely to respond to a particular treatment. This ability would allow optimal treatment to be initiated sooner, thereby potentially reducing the costs and the risks to patients and preventing radiological progression.

The search continues for biomarkers and molecular networks that can help us better understand the variable response to targeted therapy. Today, the key challenge facing rheumatologists is how best to integrate the advanced therapies into daily practice.

## Abbreviations

ACR: American College of Rheumatology; ACR20: American College of Rheumatology 20% improvement criteria; APRIL: a proliferation-inducing ligand; AS: ankylosing spondylitis; DAS: Disease Activity Score; DMARD: disease-modifying antirheumatic drug; HLA: human leucocyte antigen; IFN: interferon; IL: interleukin; MTX: methotrexate; NF-κB: nuclear factor-κB; PsA: psoriatic arthritis; RA: rheumatoid arthritis; RCT: randomised controlled trial; TNF: tumour necrosis factor.

## Competing interests

PPT has served as a consultant to Abbott, BMS, Merck-Serono, Pfizer, Roche, Schering-Plough and Wyeth. JRK has served as a consultant to Wyeth for Europe, and he lectures on behalf of Abbott, Pfizer, Roche and Wyeth.

## References

[B1] Tracey D Klareskog L Sasso EH Salfeld JG Tak PP Tumor necrosis factor antagonist mechanisms of action: a comprehensive review Pharmacol Ther 2008 117 244 279 10.1016/j.pharmthera.2007.10.001 18155297

[B2] Evangelisto A Wakefield R Emery P Imaging in early arthritis Best Pract Res Clin Rheumatol 2004 18 927 943 10.1016/j.berh.2004.07.002 15501190

[B3] Combe B Landewe R Lukas C Bolosiu HD Breedveld F Dougados M Emery P Ferraccioli G Hazes JM Klareskog L Machold K Martin-Mola E Nielsen H Silman A Smolen J Yazici H EULAR recommendations for the management of early arthritis: report of a task force of the European Standing Committee for International Clinical Studies Including Therapeutics (ESCISIT) Ann Rheum Dis 2007 66 34 45 16396980 10.1136/ard.2005.044354 PMC1798412

[B4] American College of Rheumatology Subcommittee on Rheumatoid Arthritis Guidelines Guidelines for the management of rheumatoid arthritis: 2002 update Arthritis Rheum 2002 46 328 346 11840435 10.1002/art.10148

[B5] Feldmann M Brennan FM Maini RN Rheumatoid arthritis Cell 1996 85 307 310 10.1016/S0092-8674(00)81109-5 8616886

[B6] Braun J Baraliakos X Brandt J Sieper J Therapy of ankylosing spondylitis. Part II: biological therapies in the spondyloarthritides Scand J Rheumatol 2005 34 178 190 10.1080/03009740510026599 16134723

[B7] Veale DJ Ritchlin C FitzGerald O Immunopathology of psoriasis and psoriatic arthritis Ann Rheum Dis 2005 64 Suppl 2 ii26 ii29 15708930 10.1136/ard.2004.031740 PMC1766860

[B8] Choy EHS Panayi GS Cytokine pathways and joint inflammation in rheumatoid arthritis N Engl J Med 2001 344 907 916 10.1056/NEJM200103223441207 11259725

[B9] Enbrel (Etanercept) Summary of Product Characteristics http://www.medicines.org.uk/emc/medicine/19161/SPC/Enbrel%2025mg%20solution%20for%20injection%20in%20pre-filled%20syringe

[B10] Calabrese LH Molecular differences in anticytokine therapies Clin Exp Rheumatol 2003 21 241 248 12747285

[B11] Remicade (Infliximab) Summary of Product Characteristics http://www.medicines.org.uk/EMC/medicine/3236/SPC/Remicade+100mg+powder+for+concentrate+for+solution+for+infusion

[B12] Humira (Adalimumab) Summary of Product Characteristics http://www.medicines.org.uk/emc/medicine/21201/SPC/Humira%20Pen%20and%20Syringe

[B13] Emery P Breedveld FC Hall S Durez P Chang DJ Robertson D Singh A Pedersen RD Koenig AS Freundlich B Comparison of methotrexate monotherapy with a combination of methotrexate and etanercept in active, early, moderate to severe rheumatoid arthritis (COMET): a randomised, double-blind, parallel treatment trial Lancet 2008 372 375 382b 10.1016/S0140-6736(08)61000-4 18635256

[B14] Goekoop-Ruiterman YP de Vries-Bouwstra JK Allaart CF van Zeben D Kerstens PJ Hazes JM Zwinderman AH Ronday HK Han KH Westedt ML Gerards AH van Groenendael JH Lems WF van Krugten MV Breedveld FC Dijkmans BA Clinical and radiographic outcomes of four different treatment strategies in patients with early rheumatoid arthritis (the BeSt study): a randomized, controlled trial Arthritis Rheum 2005 52 3381 3390 10.1002/art.21405 16258899

[B15] Breedveld FC Weisman MH Kavanaugh AF Cohen SB Pavelka K van Vollenhoven R Sharp J Perez JL Spencer-Green GT The PREMIER study: a multicenter, randomized, double-blind clinical trial of combination therapy with adalimumab plus methotrexate versus methotrexate alone or adalimumab alone in patients with early aggressive rheumatoid arthritis who had not had previous methotrexate treatment Arthritis Rheum 2006 54 26 37 10.1002/art.21519 16385520

[B16] Hochberg MC Lebwohl MG Plevy SE Hobbs KF Yocum DE The benefit/risk profile of TNF-blocking agents: findings of a consensus panel Semin Arthritis Rheum 2005 34 819 836 10.1016/j.semarthrit.2004.11.006 15942917

[B17] Schiff MH Burmester GR Kent JD Pangan AL Kupper H Fitzpatrick SB Donovan C Safety analysis of adalimumab (HUMIRA*) in global clinical trials and US postmarketing surveillance of patients with rheumatoid arthritis Ann Rheum Dis 2006 65 889 894 16439435 10.1136/ard.2005.043166 PMC1798196

[B18] Saag KG Teng GG Patkar NM Anuntiyo J Finney C Curtis JR Paulus HE Mudano A Pisu M Elkins-Melton M Outman R Allison JJ Suarez Almazor M Bridges SL Jr Chatham WW Hochberg M MacLean C Mikuis T Moreland LW O'Dell J Turkiewicz AM Furst DE American College of Rheumatology American College of Rheumatology 2008 recommendations for the use of nonbiologic and biologic disease-modifying antirheumatic drugs in rheumatoid arthritis Arthritis Rheum 2008 59 762 784 10.1002/art.23721 18512708

[B19] Bellofiore B Matarese A Balato N Gaudiello F Scarpa R Atteno M Bocchino M Sanduzzi A Prevention of tuberculosis in patients taking tumor necrosis factor-alpha blockers J Rheumatol 2009 83 76 77 10.3899/jrheum.090233 19661550

[B20] Dixon WG Hyrich KL Watson KD Lunt M Galloway J Ustianowski A BSRBR Control Centre Consortium Symmons DPM the BSR Biologics Register Drug-specific risk of tuberculosis in patients with rheumatoid arthritis treated with anti-TNF therapy: results from the British Society for Rheumatology Biologics Register (BSRBR) Ann Rheum Dis 2010 69 522 528 10.1136/ard.2009.118935 19854715 PMC2927681

[B21] Askling J Fored CM Brandt L Baecklund E Bertilsson L Cöster L Geborek P Jacobsson LT Lindblad S Lysholm J Rantapää-Dahlqvist S Saxne T Romanus V Klareskog L Feltelius N Risk and case characteristics of tuberculosis in rheumatoid arthritis associated with tumor necrosis factor antagonists in Sweden Arthritis Rheum 2005 52 1986 1992 10.1002/art.21137 15986370

[B22] Tubach F Salmon D Ravaud P Allanore Y Goupille P Bréban M Pallot-Prades B Pouplin S Sacchi A Chichemanian RM Bretagne S Emilie D Lemann M Lorthololary O Mariette X RATIO group Risk of tuberculosis is higher with anti-tumor necrosis factor monoclonal antibody therapy than with soluble tumor necrosis factor receptor therapy: the three-year prospective French Research Axed on Tolerance of Biotherapies registry Arthritis Rheum 2009 60 1884 1894 10.1002/art.24632 19565495 PMC2921546

[B23] Ellerin T Rubin RH Weinblatt ME Infections and anti-tumor necrosis factor α therapy Arthritis Rheum 2003 48 3013 3022 10.1002/art.11301 14613261

[B24] Bartelds GM Wijbrandts CA Nurmohamed MT Stapel S Lems WF Aarden L Dijkmans BA Tak PP Wolbink GJ Clinical response to adalimumab: relationship to anti-adalimumab antibodies and serum adalimumab concentrations in rheumatoid arthritis Ann Rheum Dis 2007 66 921 926 10.1136/ard.2006.065615 17301106 PMC1955110

[B25] Wolbink GJ Vis M Lems W Voskuyl AE de Groot E Nurmohamed MT Stapel S Tak PP Aarden L Dijkmans B Development of anti-infliximab antibodies and relationship to clinical response in patients with rheumatoid arthritis Arthritis Rheum 2006 54 711 715 10.1002/art.21671 16508927

[B26] van Kuijk AW de Groot M Stapel SO Dijkmans BA Wolbink GJ Tak PP Relationship between the clinical response to adalimumab treatment and serum levels of adalimumab and anti-adalimumab antibodies in patients with psoriatic arthritis Ann Rheum Dis 2010 69 624 625 10.1136/ard.2009.108787 20223840

[B27] de Vries MK van der Horst-Bruinsma IE Nurmohamed MT Aarden LA Stapel SO Peters MJ van Denderen JC Dijkmans BA Wolbink GJ Immunogenicity does not influence treatment with etanercept in patients with ankylosing spondylitis Ann Rheum Dis 2009 68 531 535 10.1136/ard.2008.089979 18375542

[B28] de Vries MK Wolbink GJ Stapel SO de Vrieze H van Denderen JC Dijkmans BA Aarden LA van der Horst-Bruinsma IE Decreased clinical response to infliximab in ankylosing spondylitis is correlated with anti-infliximab formation Ann Rheum Dis 2007 66 1252 1254 10.1136/ard.2007.072397 17472991 PMC1955152

[B29] Zou J Rudwaleit M Brandt J Thiel A Braun J Sieper J Down-regulation of the non-specific and antigen-specific T cell cytokine response in ankylosing spondylitis during treatment with infliximab Arthritis Rheum 2003 48 780 790 10.1002/art.10847 12632433

[B30] Zou J Rudwaleit M Brandt J Thiel A Braun J Sieper J Up regulation of the production of tumour necrosis factor alpha and interferon gamma by T cells in ankylosing spondylitis during treatment with etanercept Ann Rheum Dis 2003 62 561 564 10.1136/ard.62.6.561 12759295 PMC1754568

[B31] Wijbrandts CA Remans PH Klarenbeek PL Wouters D van den Bergh Weerman MA Smeets TJ Vervoordeldonk MJ Baeten D Tak PP Analysis of apoptosis in peripheral blood and synovial tissue very early after initiation of infliximab treatment in rheumatoid arthritis patients Arthritis Rheum 2008 58 3330 3339 10.1002/art.23989 18975323

[B32] Scallon B Cai A Solowski N Rosenberg A Song XY Shealy D Wagner C Binding and functional comparisons of two types of tumor necrosis factor antagonists J Pharmacol Exp Ther 2002 301 418 426 10.1124/jpet.301.2.418 11961039

[B33] Smeets TJ Kraan MC van Loon ME Tak PP Tumor necrosis factor alpha blockade reduces the synovial cell infiltrate early after initiation of treatment, but apparently not by induction of apoptosis in synovial tissue Arthritis Rheum 2003 48 2155 2162 10.1002/art.11098 12905468

[B34] Shergy WJ Isern RA Cooley DA Harshbarger JL Huffstutter JE Hughes GM Spencer-Smith EA Goldman AL Roth SH Toder JS Warner D Quinn A Keenan GF Schaible TF PROMPT Study Group Profiling Remicade Onset with MTX in a Prospective Trial: open label study to assess infliximab safety and timing of onset of clinical benefit among patients with rheumatoid arthritis J Rheumatol 2002 29 667 677 11950005

[B35] Charles P Elliott MJ Davis D Potter A Kalden JR Antoni C Breedveld FC Smolen JS Eberl G de Woody K Feldmann M Maini RN Regulation of cytokines, cytokine inhibitors, and acute-phase proteins following anti-TNF-α therapy in rheumatoid arthritis J Immunol 1999 163 1521 1528 10415055

[B36] Smolen JS Han C Bala M Maini RN Kalden JR van der Heijde D Breedveld FC Furst DE Lipsky PE ATTRACT Study Group Evidence of radiographic benefit of treatment with infliximab plus methotrexate in rheumatoid arthritis patients who had no clinical improvement: a detailed subanalysis of data from the anti-turn or necrosis factor trial in rheumatoid arthritis with concomitant therapy study Arthritis Rheum 2005 52 1020 1030 10.1002/art.20982 15818697

[B37] Hermann J Mueller T Fahrleitner A Dimai HP Early onset and effective inhibition of bone resorption in patients with rheumatoid arthritis treated with the tumour necrosis factor alpha antibody infliximab Clin Exp Rheumatol 2003 21 473 476 12942699

[B38] Den Broeder A van de Putte L Rau R Schattenkirchner M Van Riel P Sander O Binder C Fenner H Bankmann Y Velagapudi R Kempeni J Kupper H A single-dose, placebo-control led study of the fully human anti-turn or necrosis factor-alpha antibody adalimumab (D2E7) in patients with rheumatoid arthritis J Rheumatol 2002 29 2288 2298 12415583

[B39] Tak PP Rigby WF Rubbert-Roth A Peterfy CG van Vollenhoven RF Stohl W Hessey E Chen A Tyrrell H Shaw TM Inhibition of joint damage and improved clinical outcomes with rituximab plus methotrexate in early active rheumatoid arthritis: the IMAGE trial Ann Rheum Dis 2011 70 39 46 10.1136/ard.2010.137703 20937671

[B40] van der Heijde D Klareskog L Rodriguez-Valverde V Codreanu C Bolosiu H Melo-Gomes J Tornero-Molina J Wajdula J Pedersen R Fatenejad S TEMPO Study Investigators Comparison of etanercept and methotrexate, alone and combined, in the treatment of rheumatoid arthritis: two-year clinical and radiographic results from the TEMPO study, a double-blind, randomized trial Arthritis Rheum 2006 54 1063 1074 10.1002/art.21655 16572441

[B41] van Vollenhoven RF Ernestam S Geborek P Petersson IF Cöster L Waltbrand E Zickert A Theander J Thörner A Hellström H Teleman A Dackhammar C Akre F Forslind K Ljung L Oding R Chatzidionysiou A Wörnert M Bratt J Addition of infliximab compared with addition of sulfasalazine and hydroxychloroquine to methotrexate in patients with early rheumatoid arthritis (Swefot trial): 1-year results of a randomized trial Lancet 2009 374 459 466 10.1016/S0140-6736(09)60944-2 19665644

[B42] Smolen JS Landewé R Breedveld FC Dougados M Emery P Gaujoux-Viala C Gorter S Knevel R Nam J Schoels M Aletaha D Buch M Gossec L Huizinga T Bijlsma JW Burmester G Combe B Cutolo M Gabay C Gomez-Reino J Kouloumas M Kvien TK Martin-Mola E Mclnnes I Pavelka K van Riel P Scholte M Scott DL Sokka T Valesini G EULAR recommendations for the management of rheumatoid arthritis with synthetic and biological disease-modifying antirheumatic drugs Ann Rheum Dis 2010 69 964 975 10.1136/ard.2009.126532 20444750 PMC2935329

[B43] Hetland ML Christensen IJ Tarp U Dreyer L Hansen A Hansen IT Kollerup G Linde L Lindegaard HM Poulsen UE Schlemmer A Jensen DV Jensen S Hostenkamp G Østergaard M All Departments of Rheumatology in Denmark Direct comparison of treatment responses, remission rates, and drug adherence in patients with rheumatoid arthritis treated with adalimumab, etanercept, or infliximab: results from eight years of surveillance of clinical practice in the nationwide Danish DANBIO registry Arthritis Rheum 2010 62 22 32 10.1002/art.27227 20039405

[B44] Hyrich K Symmons D Watson K Silman A Baseline comorbidity levels in biologic and standard DMARD treated patients with rheumatoid arthritis: results from a national patient register Ann Rheum Dis 2006 65 895 898 16339291 10.1136/ard.2005.043158 PMC1798204

[B45] Oldroyd J Schachna L Buchbinder R Staples M Murphy B Bond M Briggs A Lassere M March L Ankylosing spondylitis patients commencing biologic therapy have high baseline levels of comorbidity: a report from the Australian Rheumatology Association Database Int J Rheumatol 2009 open access article available at http://www.hindawi.com/journals/ijr/2009/268569/10.1155/2009/268569 PMC2809318 20107564

[B46] Zink A Strangfeld A Schneider M Herzer P Hierse F Stoyanova-Scholz M Wassenberg S Kapelle A Listing J Effectiveness of tumor necrosis factor inhibitors in rheumatoid arthritis in an observational cohort study: comparison of patients according to their eligibility for major randomized clinical trials Arthritis Rheum 2006 54 3399 3407 10.1002/art.22193 17075823

[B47] Kievit W Fransen J Oerlemans AJ KLiper HH van der Laar MA de Rooij DJ De Gendt CM Ronday KH Jansen TL van Oijen PC Bius HL Adang EM van Riel PL The efficacy of anti-TNF in rheumatoid arthritis: a comparison between randomised controlled trials and clinical practice Ann Rheum Dis 2007 66 1473 1478 10.1136/ard.2007.072447 17426065 PMC2111629

[B48] Østergaard M Unkerskov J Linde L Krogh NS Ravn T Ringsdal VS Petri A Andersen LS Tarp U Hansen A Hjardem E Hetland ML Low remission rates but long drug survival in rheumatoid arthritis patients treated with infliximab or etanercept: results from the nationwide Danish DANBiO database Scand J Rheu matoí 2007 36 151 154 10.1080/03009740601089267 17476624

[B49] Klinkhoff A Biological agents for rheumatoid arthritis: targeting both physical function and structural damage Drugs 2004 64 1267 1283 10.2165/00003495-200464120-00001 15200343

[B50] van der Heijde D Kavanaugh A Gladman DD Antoni C Krueger GG Guzzo C Zhou B Dooley LT de Vlam K Geusens P Birbara C Halter D Beutler A Infliximab inhibits progression of radiographic damage in patients with active psoriatic arthritis through one year of treatment. Results from the induction and maintenance psoriatic arthritis clinical trial 2 Arthritis Rheum 2007 56 2698 2707 10.1002/art.22805 17665424

[B51] Quinn MA Conaghan PG O'Connor PI Karim Z Greenstein A Brown A Brown C Fraser A Jarret S Emery P Very early treatment with infliximab in addition to methotrexate in early, poor-prognosis rheumatoid arthritis reduces magnetic resonance imaging evidence of synovitis and damage, with sustained benefit after infliximab withdrawal. Results from a twelvemonth randomized, double-blind, placebo-controlled trial Arthritis Rheum 2005 52 27 35 10.1002/art.20712 15641102

[B52] van der Kooij SM le Cessie S Goekoop-Ruiterman YP de Vries-Bouwstra JK van Zeben D Kerstens PI Hazes JM van Schaardenburg D Breedveld FC Dijkmans BA Allaart CF Clinical and radiological efficacy of initial vs delayed treatment with infliximab plus methotrexate in patients with early rheumatoid arthritis Ann Rheum Dis 2009 68 1153 1158 10.1136/ard.2008.093294 18930988

[B53] Klarenbeek NB Guler-Yuksel M van der Kooij SM van der Heijde DMFM Huizinga TWJ Kerstens PJSM Peeters AJ Ronday HK Westedt ML Dijkmans BAC Allart CF Clinical outcomes of four different treatment strategies in patients with recent-onset rheumatoid arthritis: 5-years results of the BeSt study [abstract] Ann Rheum Dis 2008 67 suppl II 187

[B54] Barkham N Keen HI Coates LC O'Connor P Hensor E Fraser AD Cawkwell LS Bennett A McGonagle D Emery P Clinical and imaging efficacy of infliximab in HLA-B27-positive patients with magnetic resonance imaging-determined early sacroiliitis Arthritis Rheum 2009 60 946 954 10.1002/art.24408 19333933

[B55] Hyrich K Watson KD Silman AJ Symmons DPM British Society for Rheumatology Biologics Register Predictor of response to anti-TNF-α therapy among patients with rheumatoid arthritis: results from the British Society for Rheumatology Biologics Register Rheumatology (Oxford) 2006 45 1558 1565 10.1093/rheumatology/kel149 16705046

[B56] Finckh A Simard JF Gabay C Guerne PA Swiss Clinical Quality Management of RA Physicians Evidence for differential acquired drug resistance to anti-tumour necrosis factor agents in rheumatoid arthritis Ann Rheum Dis 2006 65 746 752 10.1136/ard.2005.045062 16339288 PMC1798167

[B57] Carmona L Gómez-Reino JJ Survival of TNF antagonists in spondylarthritis is better than in rheumatoid arthritis. Data from the Spanish registry BIOBADASER Arthritis Res Ther 2006 8 R72 10.1186/ar1941 16620398 PMC1526631

[B58] Wijbrandts CA Dijkgraaf MG Kraan MC Vinkenoog M Smeets TJ Dinant H Vos K Lems WF Wolbink GJ Sijpkens D Dijkmans BA Tak PP The clinical response to infliximab in rheumatoid arthritis is in part dependent on pre-treatment tumour necrosis factor alpha expression in the synovium Ann Rheum Dis 2008 67 1139 1144 10.1136/ard.2007.080440 18055470 PMC2564801

[B59] Klaasen R Thurlings RM Wijbrandts CA van Kuijk AW Baeten D Gerlag DM Tak PP The relationship between synovial lymphocyte aggregates and the clinical response to infliximab in rheumatoid arthritis: a prospective study Arthritis Rheum 2009 60 3217 3224 10.1002/art.24913 19877042

[B60] Blom M Kievit W Fransen J Kuper IH den Broeder AA De Gendt CM Jansen TL Bius HL van de Laar MA van Riel PL The reason for discontinuation of the first tumor necrosis factor (TNF) blocking agent does not influence the effect of a second TNF blocking agent in patients with rheumatoid arthritis J Rheumatol 2009 36 2171 2177 10.3899/jrheum.090054 19723902

[B61] van Vollenhoven R Harju A Brannemark S Klareskog L Treatment with infliximab (Remicade) when etanercept (Enbrel) has failed or vice versa: data from the STURE registry showing that switching tumour necrosis factor alpha blockers can make sense Ann Rheum Dis 2003 62 1195 1198 10.1136/ard.2003.009589 14644858 PMC1754380

[B62] van Vollenhoven RF Switching between anti-tumour necrosis factors: trying to get a handle on a complex issue Ann Rheum Dis 2007 66 849 851 10.1136/ard.2007.069872 17576784 PMC1955116

[B63] Keystone EC Switching tumor necrosis factor inhibitors: an opinion Nat Clin Pract Rheumatol 2006 2 576 577 10.1038/ncprheum0339 17075592

[B64] Agarwal SK Glass RJ Shadick NA Coblyn JS Anderson RJ Maher NE Weinblatt ME Solomon DH Predictors of discontinuation of tumor necrosis factor inhibitors in patients with rheumatoid arthritis J Rheumatol 2008 35 1737 1744 18634159 PMC2756035

[B65] Feldmann M Development of anti-TNF therapy for rheumatoid arthritis Nat Rev Immunol 2002 2 364 371 10.1038/nri802 12033742

[B66] Voulgari PV Emerging drugs for rheumatoid arthritis Expert Opin Emerging Drugs 2008 13 175 196 10.1517/14728214.13.1.175 18321156

[B67] Noss EH Brenner MB The role and therapeutic implications of fibroblastlike synoviocytes in inflammation and cartilage erosion in rheumatoid arthritis Immunol Rev 2008 223 252 270 10.1111/j.1600-065X.2008.00648.x 18613841

[B68] Mclnnes IB Schett G Cytokines in the pathogenesis of rheumatoid arthritis Nat Rev Immunol 2007 7 429 442 10.1038/nri2094 17525752

[B69] Ralph JA Morand EF MAPK phosphatases as novel targets for rheumatoid arthritis Expert Opin Ther Targets 2008 12 795 808 10.1517/14728222.12.7.795 18554149

[B70] Koczan D Drynda S Hecker M Drynda A Guthke R Kekow J Thiesen HJ Molecular discrimination of responders and nonresponders to anti-TNFα therapy in rheumatoid arthritis by etanercept Arthritis Res Ther 2008 10 R50 18454843 10.1186/ar2419 PMC2483439

[B71] Tas SW Vervoordeldonk MJ Tak PP Gene therapy targeting nuclear factor-KB: towards clinical application in inflammatory diseases and cancer Curr Gene Ther 2009 9 160 170 10.2174/156652309788488569 19519361 PMC2864453

[B72] Dörner T Kinnman N Tak PP Targeting B cells in immune-media ted inflammatory disease: a comprehensive review of mechanisms of action and identification of biomarkers Pharmacol Ther 2010 125 464 475 10.1016/j.pharmthera.2010.01.001 20097226

[B73] Vischer TL Werner-Favre CF Wen L Zubler RH Quantitative analysis of precursors frequency of rheumatoid factor (RF) producing human B cells Scand J Rheumatol Suppl 1988 75 123 126 3266359 10.3109/03009748809096752

[B74] Tak PP Thurlings RM Rossier C Nestorov I Dimic A Mircetic V Rischmueller M Nasonov E Shmidt E Emery P Munafo A Atacicept in patients with rheumatoid arthritis: results of a multicenter, phase lb, double-blind, placebo-controlled, dose-escalating, single-and repeated-dose study Arthritis Rheum 2008 58 61 72 10.1002/art.23178 18163485

[B75] Youinou P Jamin C Saraux A B-cell: a logical target for treatment of rheumatoid arthritis Clin Exp Rheumatol 2007 25 318 328 17543163

[B76] Youinou P Jamin C Pers JO Berthou C Saraux A Renaudineau Y B-lymphocytes are required for development and treatment of autoimmune diseases Ann N Y Acad Sci 2005 1050 19 33 10.1196/annals.1313.003 16014517

[B77] Chaiamnuay S Bridges SL Jr The role of B cells and autoantibodies in rheumatoid arthritis Pathophysiology 2005 12 203 216 10.1016/j.pathophys.2005.07.007 16102949

[B78] Tak PP Chemokine inhibition in inflammatory arthritis Best Pract Res Clin Rheumatol 2006 20 929 939 10.1016/j.berh.2006.06.001 16980215

[B79] Schett G Review: immune cells and mediators of inflammatory arthritis Autoimmunity 2008 41 224 229 10.1080/08916930701694717 18365836

[B80] Bartelds GM Wijbrandts CA Nurmohamed MT Stapel SO Lems WF Aarden L Dijkmans BA Tak PP Wolbink GJ Anti-infliximab and anti-adalimumab antibodies in relation to response to adalimumab in infliximab switchers and anti-TNF naive patients: a cohort study Ann Rheum Dis 2010 69 817 821 10.1136/ard.2009.112847 19581278

[B81] Coenen MJ Toonen EJ Scheffer H Radstake TR Barrera P Franke B Pharmacogenetics of anti-TNF treatment in patients with rheumatoid arthritis Pharmacogenomics 2007 8 761 773 10.2217/14622416.8.7.761 17638513

[B82] Cohen SB Emery P Greenwald MW Dougados M Furie RA Genovese MC Keystone EC Loveless JE Burmester GR Cravets MW Hessey EW Shaw T Totoritis MC REFLEX Trial Group Rituximab for rheumatoid arthritis refractory to anti-tumor necrosis factor therapy: results of a multicenter, randomized, double-blind, placebo-controlled, phase III trial evaluating primary efficacy and safety at twenty-four weeks Arthritis Rheum 2006 54 2793 2806 10.1002/art.22025 16947627

[B83] MabThera (Rituximab) Summary of Product Characteristics http://www.medicines.org.uk/EMC/medicine/2570/SPC/Mabthera+100mg+and+500mg+concentrate+for+solution+for+infusion

[B84] Keystone E Emery P Peterfy CG Tak PP Cohen S Genovese MC Dougados M Burmester GR Greenwald M Kvien TK Williams S Hagerty D Cravets MW Shaw T Rituximab inhibits structural joint damage in patients with rheumatoid arthritis with an inadequate response to tumour necrosis factor inhibitor therapies Ann Rheum Dis 2009 68 216 221 10.1136/ard.2007.085787 18388156

[B85] Finckh A Ciurea A Brulhart L Möller B Walker UA Courvoisier D Kyburz D Dudler J Gabay C Swiss Clinical Quality Management Programme for Rheumatoid Arthritis Which subgroup of patients with rheumatoid arthritis benefits from switching to rituximab versus alternative anti-tumour necrosis factor (TNF) agents after previous failure of an anti-TNF agent? Ann Rheum Dis 2010 69 387 393 10.1136/ard.2008.105064 19416802 PMC2800201

[B86] Kormelink TG Tekstra J Thurlings RM Boumans MH Vos K Tak PP Bijlsma JW Lafeber FP Redegeld FA van Roon JA Decrease in immunoglobulin free light chains in patients with rheumatoid arthritis upon rituximab (anti-CD20) treatment correlates with decrease in disease activity Ann Rheum Dis 2010 69 2137 2144 10.1136/ard.2009.126441 20679475

[B87] Keystone E Fleischmann R Emery P Furst DE van Vollenhoven R Bathon J Dougados M Baldassare A Ferraccioli G Chubick A Udell J Cravets MW Agarwal A Cooper S Magrini F Safety and efficacy of additional courses of rituximab in patients with active rheumatoid arthritis: an open-label extension analysis Arthritis Rheum 2007 56 3896 3908 10.1002/art.23059 18050221

[B88] Dixon WG Watson K Lunt M Hyrich KL Silman AJ Symmons DP British Society for Rheumatology Biologics Register Rates of serious infection, including site-specific and bacterial intracellular infection, in rheumatoid arthritis patients receiving anti-tumor necrosis factor therapy: results from the British Society for Rheumatology Biologics Register Arthritis Rheum 2006 54 2368 2376 10.1002/art.21978 16868999

[B89] Dass S Vital EM Emery P Development of psoriasis after B cell depletion with rituximab Arthritis Rheum 2007 56 2715 2718 10.1002/art.22811 17665440

[B90] Ko JM Gottlieb AB Kerbleski JF Induction and exacerbation of psoriasis with TNF-blockade therapy: a review and analysis of 127 cases J Dermatolog Treat 2009 20 100 108 10.1080/09546630802441234 18923992

[B91] Emery P Durez P Dougados M Legerton CW Becker JC Vratsanos G Genant HK Peterfy C Mitra P Overfield S Qi K Westhovens R Impact of T-cell costimulation modulation in patients with undifferentiated inflammatory arthritis or very early rheumatoid arthritis: a clinical and imaging study of abatacept (the ADJUST trial) Ann Rheum Dis 2010 69 510 516 10.1136/ard.2009.119016 19933744 PMC2927615

[B92] Orencia (Sbatacept) Summary of Product Characteristics http://www.medicines.org.uk/EMC/medicine/19714/SPC/ORENCIA+250+mg+powder+for+concentrate+for+solution+for+infusion

[B93] Voll RE Kalden JR Do we need new treatment that goes beyond tumor necrosis factor blockers for rheumatoid arthritis? Ann NY Acad Sci 2005 1051 799 810 10.1196/annals.1361.123 16127017

[B94] Schiff M Bessette L Evaluation of abatacept in biologic-naïve patients with active rheumatoid arthritis [includes some 2-year data from ATTEST trial] Clin Rheumatol 2010 29 583 591 10.1007/s10067-009-1363-0 20099018

[B95] Genant HK Peterfy CG Westhovens R Becker J-C Aranda R Vratsanos G Teng J Kremer JM Abatacept inhibits progression of structural damage in rheumatoid arthritis: results from the long-term extension of the AIM trial Ann Rheum Dis 2008 67 1084 1089 10.1136/ard.2007.085084 18086727 PMC2569144

[B96] Genovese MC Schiff M Luggen M Becker J-C Aranda R Teng J Li T Schmidely N Le Bars M Dougados M Efficacy and safety of the selective co-stimulation modulator abatacept following 2 years of treatment in patients with rheumatoid arthritis and an inadequate response to anti-tumour necrosis factor therapy Ann Rheum Dis 2008 67 547 554 17921185 10.1136/ard.2007.074773

[B97] Westhovens R Kremer J Moreland L Emery P Russell A Li T Aranda R Becker J-C Qi K Dougados M Safety and efficacy of the selective costimulation modulator abatacept in patients with rheumatoid arthritis receiving background methotrexate: a 5-year extended phase MB study J Rheumatol 2009 36 736 742 10.3899/jrheum.080813 19273451

[B98] Schiff M Keiserman M Codding C Songcharoen S Berman A Nayiager S Saidate C Li T Aranda R Becker J-C Lin C Cornet PLN Dougados M Efficacy and safety of abatacept or infliximab vs placebo in ATTEST: a phase III, multi-centre, randomised, double-blind, placebo-controlled study in patients with rheumatoid arthritis and an inadequate response to methotrexate [1-year results] Ann Rheum Dis 2008 67 1096 1103 10.1136/ard.2007.080002 18055472 PMC2564802

[B99] Schiff M Keiserman M Codding C Songcharoen S Berman A Nayiager S Saidate C Aranda R Becker JC Zhao C Le Bars M Dougados M Two-year efficacy and safety in abatacept-treated patients with RA who received continuous therapy or switched from infliximab to abatacept: the ATTEST trial [abstract] Ann Rheum Dis 2009 38 575

[B100] Schiff M Pritchard C Huffstutter JE Rodriguez-Valverde V Durez P Zhou X Li T Bahrt K Kelly S Le Bars M Genovese MC The 6-month safety and efficacy of abatacept in patients with rheumatoid arthritis who underwent a washout after anti-tumour necrosis factor therapy or were directly switched to abatacept: the ARRIVE trial Ann Rheum Dis 2009 68 1708 1714 10.1136/ard.2008.099218 19074911 PMC2756956

[B101] RoActemra (Tocilizumab) Summary of Product Characteristics http://www.medicines.org.uk/EMC/medicine/22312/PIL/RoActemra+20mg+ml+Concentrate+for+Solution+for+Infusion

[B102] Jones G The AMBITION trial: tocilizumab monotherapy for rheumatoid arthritis Expert Rev Clin Immunol 2010 6 189 195 10.1586/eci.10.2 20402381

[B103] Smolen JS Beaulieu A Rubbert-Roth A Ramos-Remus C Rovensky J Alecock E Woodworth T Alten R OPTION Investigators Effect of interleukin-6 receptor inhibition with tocilizumab in patients with rheumatoid arthritis (OPTION study): a double-blind, placebo-controlled, randomised trial Lancet 2008 371 987 997 10.1016/S0140-6736(08)60453-5 18358926

[B104] Gomez-Reino JJ Fairfax MJ Pavelka K Alecock E Woodworth T Genovese M Targeted inhibition of IL-6 signalling with tocilizumab improves quality of life and function in patients with rheumatoid arthritis with inadequate response to a range of DMARDs [abstract] Arthritis Rheum 2007 56 12 4234

[B105] Emery P Keystone E Tony H Cantagrel A van Vollenhoven R Sanchez A Alecock E Lee J Kremer J IL-6 receptor inhibition with tocilizumab improves treatment outcomes in patients with rheumatoid arthritis refractory to anti-tumour necrosis factor biologicals: results from a 24-week multicentre randomised placebo-control led trial [RADIATE study] Ann Rheum Dis 2008 67 1516 1523 18625622 10.1136/ard.2008.092932 PMC3811149

[B106] Nishimoto N Interleukin-6 as a therapeutic target in candidate inflammatory diseases Clin Pharmacol Ther 2010 87 483 487 10.1038/clpt.2009.313 20182422

[B107] Nishimoto N Miyasaka N Yamamoto K Kawai S Takeuchi T Azuma J Kishimoto T Study of active controlled tocilizumab monotherapy for rheumatoid arthritis patients with an inadequate response to methotrexate (SATORI): significant reduction in disease activity and serum vascular endothelial growth factor by IL-6 receptor inhibition therapy Mod Rheumatol 2009 19 12 19 10.1007/s10165-008-0125-1 18979150 PMC2638601

[B108] Hashimoto J Garnero P van der Heijde D Miyasaka N Yamamoto K Kawai S Takeuchi T Yoshikawa H Nishimoto N Humanized anti-interleukin-6-receptor antibody (tocilizumab) monotherapy is more effective in slowing radiographic progression in patients with rheumatoid arthritis at high baseline risk for structural damage evaluated with levels of biomarkers, radiography, and BMI: data from the SAMURAI study Mod Rheumatol 2011 Epub ahead of print 10.1007/s10165-010-0325-3 PMC3036807 20574648

[B109] Kremer JL Blanco R Brzosko M Burgos-Vargas R Halland AM Vernon E Ambs P Fleischmann R Tocilizumab inhibits structural joint damage in rheumatoid arthritis patients with inadequate responses to methotrexate at 1 year: the LITHE study Arthritis Rheum 2011 63 609 621 10.1002/art.30158 21360490

[B110] Fleischmann R Vencovsky J van Vollenhoven RF Borenstein D Box J Coteur G Goel N Brezinschek HP Innes A Strand V Efficacy and safety of certolizumab pegol monotherapy every 4 weeks in patients with rheumatoid arthritis failing previous disease-modifying antirheumatic therapy: the FAST4WARD study Ann Rheum Dis 2009 68 805 811 10.1136/ard.2008.099291 19015206 PMC2674555

[B111] Smolen J Landewé RB Mease P Brzezicki J Mason D Luijtens K van Vollenhoven RF Kavanaugh A Schiff M Burmester GR Strand V Vencovsky J van der Heijde D Efficacy and safety of certolizumab pegol plus methotrexate in active rheumatoid arthritis: the RAPID 2 study. A randomised controlled trial Ann Rheum Dis 2009 68 797 804 10.1136/ard.2008.101659 19015207 PMC2674556

[B112] Kay J Matteson EL Dasgupta B Nash P Durez P Hall S Hsia EC Han J Wagner C Xu Z Visvanathan S Rahman MU Golimumab in patients with active rheumatoid arthritis despite treatment with methotrexate: a randomized, double-blind, placebo-control led, dose-ranging study Arthritis Rheum 2008 58 964 975 10.1002/art.23383 18383539

[B113] Keystone EC Genovese MC Klareskog L Hsia EC Hall ST Miranda PC Pazdur J Bae SC Palmer W Zrubek J Wiekowski M Visvanathan S Wu Z Rahman MU Golimumab, a human antibody to tumour necrosis factor α given by monthly subcutaneous injections, in active rheumatoid arthritis despite methotrexate therapy: the GO-FORWARD Study Ann Rheum Dis 2009 68 789 796 10.1136/ard.2008.099010 19066176 PMC2674549

[B114] Smolen J Kay J Doyle MK Landewe R Matteson EL Wollenhaupt J Gaylis N Murphy F Neal J Zamani O Zhou Y Visvanathan S Hsia EC Rahman MU GO-AFTER Study Investigators Golimumab in patients with active rheumatoid arthritis after treatment with tumour necrosis factor α inhibitors (GO-AFTER study): a multicentre, randomised, double-blind, placebo-controlled, phase III trial Lancet 2009 374 210 221 10.1016/S0140-6736(09)60506-7 19560810

[B115] Emery P Fleischmann RM Moreland LW Hsia EC Strusberg I Durez P Nash P Amante E Churchill M Park W Pons-Estel B Xu W Rahman MU Golimumab, a human anti-tumor necrosis factor α monoclonal antibody, injected subcutaneously every four weeks in methotrexate-naive patients with active rheumatoid arthritis [GO-BEFORE study] Arthritis Rheum 2009 60 2272 2283 10.1002/art.24638 19644849

[B116] Kavanaugh A Mclnnes I Mease P Krueger GG Gladman D Gomez-Reino J Papp K Zrubek J Mudivarthy S Mack M Visvanathan S Beutler A Golimumab, a new human tumor necrosis factor α antibody, administered every four weeks as a subcutaneous injection in psoriatic arthritis: twenty-four-week efficacy and safety results of a randomized, placebo-controlled study Arthritis Rheum 2009 60 976 986 10.1002/art.24403 19333944

[B117] Braun J Davis JC van der Heijde D Deodhar A Diekman L Sieper J Kim SII Mack M Han J Hsu B Beutler A Inman R Golimumab, a new, human, TNF-alpha antibody administered subcutaneously every 4 weeks, in ankylosing spondylitis (AS): 24-week efficacy and safety results of the randomized, placebo-controlled GO-RAISE study [abstract] Ann Rheum Dis 2008 67 Suppl II 58

[B118] Inman RD Davis JC Jr van der Heijde D Diekman L Sieper J Kim SI Mack M Han J Visvanathan S Xu Z Hsu B Beutler A Braun J Efficacy and safety of golimumab in patients with ankylosing spondylitis: results of a randomized, double-blind, placebo-controlled, phase III trial [GO-RAISE] Arthritis Rheum 2008 58 3402 3412 10.1002/art.23969 18975305

[B119] Gottlieb A Korman NJ Gordon KB Feldman SR Lebwohl M Koo JY Van Voorhees AS Elmets CA Leonardi CL Beutner KR Bhushan R Menter A Guidelines of care for the management of psoriasis and psoriatic arthritis. Section 2. Psoriatic arthritis: overview and guidelines of care for treatment with an emphasis on the biologics J Am Acad Dermatol 2008 58 851 864 10.1016/j.jaad.2008.02.040 18423261

[B120] Kremer JM Bloom BJ Breedveld FC Coombs JH Fletcher MP Gruben D Krishnaswami S Burgos-Vargas R Wilkinson B Zerbini CAF Zwillich SH The safety and efficacy of a JAK inhibitor in patients with active rheumatoid arthritis: results of a double-blind, placebo-controlled phase lla trial of three dosage levels of CP-690,550 versus placebo Arthritis Rheum 2009 60 1895 1905 10.1002/art.24567 19565475

[B121] Coombs JH Bloom BJ Breedveld FC Fletcher MP Gruben D Kremer JM Burgos-Vargas R Wilkinson B Zerbini CA Zwillich SH Improved pain, physical functioning and health status in patients with rheumatoid arthritis treated with CP-690,550, an orally active Janus kinase (JAK) inhibitor: results from a randomised, double-blind, placebo-controlled trial Ann Rheum Dis 2010 69 413 416 10.1136/ard.2009.108159 19587388

[B122] Bahjat FR Pine PR Reitsma A Cassafer G Baluom M Grillo S Chang B Zhao FF Payan DG Grossbard EB Daikh DI An orally bioavaliable spleen tyrosine kinase inhibitor delays disease progression and prolongs survival in murine lupus Arthritis Rheum 2008 58 1433 1444 10.1002/art.23428 18438845

[B123] Pine PR Chang B Schoettler N Banquerigo ML Wang S Lau A Zhao F Grossbard EB Payan DG Brahn E Inflammation and bone erosion are suppressed in models of rheumatoid arthritis following treatment with a novel Syk inhibitor Clin Immunol 2007 124 244 257 10.1016/j.clim.2007.03.543 17537677

[B124] Weinblatt ME Kavanaugh A Burgos-Vargas R Dikranian AH Medrano-Ramirez G Morales-Torres JL Murphy FT Musser TK Straniero N Vicente-Gonzales AV Grossbard E Treatment of rheumatoid arthritis with a Syk kinase inhibitor: a twelve-week, randomized, placebo-controlled trial Arthritis Rheum 2008 58 3309 3318 10.1002/art.23992 18975322

[B125] Smolen JS Aletaha D Bijlsma JW Breedveld FC Boumpas D Burmester G Combe B Cutolo M de Wit M Dougados M Emery P Gibofsky A Gomez-Reino JJ Haraoui B Kalden J Keystone EC Kvien TK Mclnnes I Martin-Mola E Montecucco C Schoels M van der Heijde D T2T Expert Committee Treating rheumatoid arthritis to target: recommendations of an international task force Ann Rheum Dis 2010 69 631 637 10.1136/ard.2009.123919 20215140 PMC3015099

